# The impact of a decision aid about heart disease prevention on patients' discussions with their doctor and their plans for prevention: a pilot randomized trial

**DOI:** 10.1186/1472-6963-6-121

**Published:** 2006-09-27

**Authors:** Stacey L Sheridan, John Shadle, Ross J Simpson, Michael P Pignone

**Affiliations:** 1 Division of General Medicine and Clinical Epidemiology, University of North Carolina, Chapel Hill, NC, USA; 2 Division of Cardiology, University of North Carolina, Chapel Hill, NC, USA

## Abstract

**Background:**

Low utilization of effective coronary heart disease (CHD) prevention strategies may be due to many factors, but chief among them is the lack of patient involvement in prevention decisions. We undertook this study to test the effectiveness of an individually-tailored, computerized decision aid about CHD on patients' discussions with their doctor and their plans for CHD prevention.

**Methods:**

We conducted a pilot randomized trial in a convenience sample of adults with no previous history of cardiovascular disease to test the effectiveness of an individually-tailored, computerized decision aid about CHD prevention against a risk factor list that patients could present to their doctor.

**Results:**

We enrolled 75 adults. Mean age was 53. 59% were female, 73% white, and 23% African-American. 66% had some college education. 43% had a 10-year CHD risk of 0–5%, 25% a risk of 6–10%, 24% a risk of 11–20%, and 5% a risk of > 20%. 78% had at least one option to reduce their CHD risk, but only 45% accurately identified the strategies best supported by evidence. 41 patients received the decision aid, 34 received usual care. In unadjusted analysis, the decision aid increased the proportion of patients who discussed CHD risk reduction with their doctor from 24% to 40% (absolute difference 16%; 95% CI -4% to +37%) and increased the proportion who had a specific plan to reduce their risk from 24% to 37% (absolute difference 13%; 95% CI -7% to +34%). In pre-post testing, the decision aid also appeared to increase the proportion of patients with plans to intervene on their CHD risk (absolute increase ranging from 21% to 47% for planned medication use and 5% to 16% for planned behavioral interventions).

**Conclusion:**

Our study confirms patients' limited knowledge about their CHD risk and effective risk reduction options and provides preliminary evidence that an individually-tailored decision aid about CHD prevention might be expected to increase patients' discussions about CHD prevention with their doctor and their plans for CHD risk reduction. These findings should be replicated in studies with a larger sample size and patients at overall higher risk of CHD.

**Trial Registration**:  ClinicalTrials.gov NCT00315978

## Background

Coronary heart disease (CHD) is the leading cause of death in the United States and significant resources are being devoted to changing patterns of CHD risk. Yet, fewer than 50% of patients are using effective CHD prevention strategies for each CHD risk factor. [1-3] Low utilization of these effective CHD prevention strategies may be due to many factors, but chief among them is the lack of patient involvement in prevention decisions. Whether providers acknowledge it or not, patients ultimately decide whether or not to adhere to CHD prevention strategies. Sub-optimal decision making occurs because patients lack critical knowledge about CHD risk factors [4,5], their overall CHD risk [6,7], and effective risk reduction strategies [8,9], and are offered prevention strategies that are discordant with their priorities. [10]

Increasing patient involvement in decision making could increase the effectiveness of decision making and lead to important reductions in the burden of CHD. Preliminary evidence suggests that involving patients in the decision making process improves their knowledge about their CHD risk factors, their global CHD risk, and their prevention options. [11] Patient involvement may also allow patients to choose prevention strategies that circumvent known barriers to CHD risk reduction, thereby increasing the likelihood of adherence to chosen options. Although offering patients choices regarding CHD prevention strategies might seem controversial, it has been suggested that an approach that uses global CHD risk and facilitates choice helps patients to match the intensity of their CHD prevention efforts with their concerns for safety, cost, and the time commitment for treatments. [12]

Decision aids, which are systematically developed tools to help patients understand and participate in medical decisions, are one means of increasing patient involvement [13] and might be useful in CHD prevention. Compared with most decision aids (which help patients make decisions where the evidence of benefit is uncertain or too close to call across a population of individuals), however, the goals of a CHD prevention decision aid would be quite different: to recommend CHD prevention and encourage a choice among equally acceptable options to accomplish it. [14] The metric of a successful decision aid on CHD prevention would, therefore, also be quite different. At the decision making level, success would be demonstrated by evidence of patients' intent to intervene on elevated CHD risk; and, at an outcomes level, success would be determined by the actual reduction in CHD risk factors or risk.

In this pilot study, we tested the effects of an individually-tailored, computerized decision aid about CHD prevention on patients' decision making. Our hypothesis was that a decision aid, which allowed patients to assess their CHD risk and weigh their preferences for one or more treatment options, would increase patients' discussions with their doctor (in an effort to obtain the medical therapy necessary to reduce CHD risk) and their plans for CHD risk reduction. Secondarily, we hypothesized that the decision aid would increase patients' perceptions that CHD prevention requires an individual decision and their desire to participate in decision making.

## Methods

**Figure 1:**
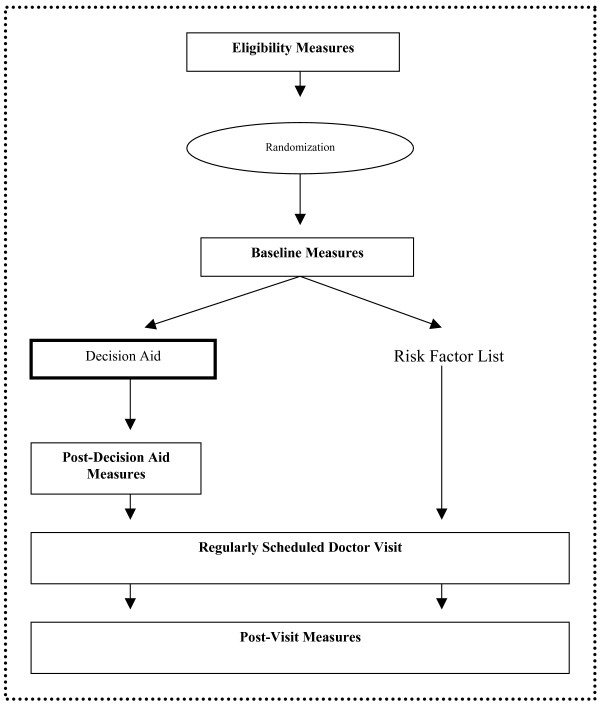
**Study design**. The dotted line margin denotes interventions and measurement occurring at a single clinical visit.

To involve patients in decision making about CHD prevention, we previously developed a decision aid that provides patients with basic information about CHD prevention and encourages patients to make individualized choices about CHD risk reduction. [15,16] In this study, we tested the effects of that decision aid against a risk factor list that patients could give to their doctor in a pilot randomized trial. Our study population, procedures, and outcomes are described in detail below. An overview of the design is depicted in Figure 1.

### Study population, recruitment, and enrollment

After approval from the University of North Carolina, School of Medicine Institutional Review Board, we recruited and enrolled a convenience sample of men and women, ages 35 to 75, who presented for care at one university internal medicine clinic. We included patients if they had no prior history of cardiovascular disease (CVD) or other serious medical condition that would limit their candidacy for screening (i.e. chronic renal failure, cirrhosis of the liver, HIV, current non-skin cancer). We excluded patients if they had participated in intensive risk factor modification through a diabetes study in our clinic or if they had no cholesterol checks in the last 3 years, limiting our ability to provide current risk estimates. We also excluded patients if they were presenting for their first visit to the clinic or they reported that they were unable to understand, speak, or read English. We identified potential participants from daily clinician schedules and, after obtaining their clinician's permission, approached them about the study in the clinic waiting room or in their exam room as they waited for their regularly scheduled visit. We then obtained informed consent from all patients who agreed to enroll in the study.

### Randomization and blinding

We used a computerized random number generator to randomize patients to receive either the Heart to Heart decision aid or a list of their CHD risk factors that they could present to their doctor. Intervention assignments were sealed in security envelopes until after subjects agreed to participate in the study. The research assistant then broke the seal to determine intervention assignment. We blinded patients to the purpose of our study by telling them only that they were participating in a study about "prevention of CHD." Doctors were not blinded and saw patients in both the decision aid and control group.

### The intervention: the Heart to Heart decision aid

After assessing baseline characteristics, we asked all patients in the intervention group to review the computerized decision aid, Heart to Heart (version 1). [15,16] Heart to Heart 1) calculates a patient's global risk of CHD events (e.g. angina, myocardial infarction, and death) in the next 10 years by combining information about their age, sex, blood pressure, total and HDL cholesterol, smoking, diabetes, and left ventricular hypertrophy (LVH) status using a continuous Framingham equation [17]; 2) provides patients with individualized information about their global CHD risk, their personal risk factors, the pros and cons of pertinent CHD risk-reducing therapies (e.g. hypertension medication, cholesterol medication, smoking cessation, and aspirin), and the risk reduction achievable after one or more therapeutic interventions; and 3) encourages patients to choose therapies that are acceptable and feasible for long-term CHD risk reduction. The tool encourages a good diet and regular exercise for everyone, but doesn't provide information on how these interventions modify CHD risk. It also provides a summary print-out that can be taken to one's visit with his or her doctor.

To assist patients in using Heart to Heart, a research assistant compiled a list of each patient's most recent risk factor information (e.g. most recent blood pressure, most recent total and HDL cholesterol, and electrocardiogram or echo-reported evidence for LVH in the last 3 years) from their medical record and provided it to the patient for use during the decision aid session. Patients then navigated the decision aid at their own speed from computers in the patient education room of our internal medicine clinic. These computers were in a private setting and equipped with a standard keyboard and mouse to facilitate individual input and response. A research assistant was available at all times to answer any questions about decision aid navigation.

### The control group

Patients randomized to the control group received only a list of their CHD risk factors that they could present to their doctor. This list included both qualitative identification of the risk factors and, where appropriate, a quantitative value for the following risk factors: blood pressure, total and HDL cholesterol, smoking, diabetes, and left ventricular hypertrophy (LVH) status.

### The provider's role

Providers saw patients for their regularly scheduled medical visit at the same time as their study related visit, but had no prescribed role in the study. Our hope was that providers would facilitate patient decisions to intervene on their CHD risk, but providers received no special training about global CHD risk, risk reduction options, or patient-provider decision making.

### Data collection

We collected self-reported data at four points in time during a single study visit: during initial eligibility assessment, at baseline, after navigation of the decision aid (intervention group only), and after the regularly scheduled provider visit. We planned this measurement strategy to capture differences between the decision aid and control groups in patients' discussions with their provider and their plans for CHD risk reduction. We secondarily planned this measurement strategy to look within the decision aid group to see whether the decision aid increased patients' perceptions that CHD prevention requires a decision and their interest in participating in decision making prior to their visit with their doctor.

### Measuring the effects of the decision aid on patient-provider discussions and patients' plans for CHD risk reduction

We measured the main effect of the decision aid on decision making in 2 ways: 1) by the proportion of patients who reported they discussed CHD risk with their doctor, and 2) by the proportion of patients that talked with their doctor who reported they had a specific plan for CHD risk reduction at the post-visit survey. We measured patient discussions with their doctor through a single question: "Did you and your doctor discuss a plan to lower your chances of having a heart attack?" Similarly, we measured plans for CHD risk reduction through a single question: "At the end of your visit, what did you decide to do, if anything, to lower your chances of heart disease?" We considered stated intent to adopt any CHD risk reducing behavior (i.e. aspirin, lipid lowering medication, anti-hypertension medication, smoking cessation medications, dietary change, or exercise) in the next 6 months as sufficient evidence of a plan for CHD risk reduction. We initially assumed that patients who didn't discuss CHD prevention with their doctor did not have a specific plan for risk reduction, but tested this assumption in subsequent analyses. Because patients who didn't talk with their doctor could have made independent plans to intervene on their CHD risk, we secondarily compared the last stated plans for each individual (regardless of whether they talked with their doctor) among patients who received the decision aid and those who did not. For instance, among those in the decision aid group who did not talk with their doctor, we considered their stated plan as the one they reported after viewing the decision aid. Similarly, among those in the control group who did not talk with their doctor, we considered their stated plan as that which they reported at baseline. There were too few patients who talked with their doctor and chose a plan to make between group comparisons regarding how the decision aid impacted choice of CHD risk reduction strategy at the end of the study. We did, however, make within group comparisons as described below.

### Measuring within group effects of the decision aid: patients' perceptions, their preferred participation in decision making, and their risk reduction choices

We measured within group effects of the decision aid using pre-post comparisons of 1) patients' perceptions that CHD prevention requires a decision, and 2) patients' desired participation in decision making. To corroborate between group differences in plans for CHD risk reduction, we also measured within group differences in patients' choices of risk reduction strategy. We assessed patients' perceptions that CHD prevention requires a decision using Likert questions assessing agreement with the following statements: "The same way of lowering the chance of heart attack *is *right for everyone" and "I am the only one who can decide how to best lower my chances of heart attack." We assessed patients' interest in participating in decision making using an adaptation of Degner's 5-item Control Preference Scale. [18] We assessed patients' plans for CHD risk reduction following the decision aid by asking "after viewing the decision aid, which of the following things (that you are not currently doing), are you NOW planning to do to lower your chances of having a heart attack?" We prompted them to choose one of the six options mentioned in the decision aid (e.g. hypertension medication, cholesterol medication, smoking cessation, aspirin, dietary change, and regular exercise) by providing these as response options, although we also allowed for write-ins using an "other" option.

### Baseline factors that might affect decision making

Because of our small sample size and the possibility that randomization may result in unequal groups, we measured several factors at baseline that might affect patients' decision making. These included the accuracy of patients' perceptions about their CHD risk, their knowledge about effective CHD prevention strategies, and their perceived barriers to risk reduction. To determine the accuracy of patients' baseline perceptions about their CHD risk, we queried them about their absolute CHD risk (e.g. 0–5%, 6–10%, 11–20%, > 20%) and then compared their self-reported risk with their actual risk, which was calculated by the Heart to Heart tool and divided into similar categories. We assessed patients' knowledge about effective CHD prevention strategies by asking them to choose effective strategies from a list of 13 possible interventions. The list included both behavioral (e.g. diet low in saturated fat, regular exercise, weight loss if overweight, smoking cessation if smoking) and medication interventions (e.g. hypertension medication, cholesterol medication, and aspirin). It also included interventions that do not reduce the chances of heart disease (e.g. estrogen, vitamin e, calcium, and monitoring morning body temperature) as distracters. As a conservative measure of overall knowledge, we calculated the proportion of patients who accurately identified all four interventions supported by the best evidence (e.g. hypertension medication, cholesterol medication, aspirin, and smoking cessation). Finally, we used a series of single questions to query patients about their perception that barriers commonly identified in the literature would prevent them from initiating CHD prevention (e.g. lack of knowledge, difficulty determining how important it is to lower CHD risk, difficulty determine the best way to lower CHD risk, concerns about other health problems, lack of symptoms, and difficulty communicating their needs to their doctor).

### Statistical analysis

To assess the success of randomization, we compared the baseline characteristics of subjects who received the decision aid with those who were in the control group. We then used Pearson's chi-square tests (SAS Statistical Software, Cary, NC) to evaluate whether the decision aid increased the proportion of patients who discussed CHD with their doctor or increased the proportion of patients with a specific plan for CHD risk reduction. We subsequently calculated the within group effects of the decision aid on 1) patients' perceptions that addressing CHD is important, 2) their perceptions that CHD prevention requires a decision, 3) their desires to participate in decision making, and 4) their stated plans for risk reduction. We calculated confidence intervals for the differences in these measures at baseline and post-decision aid using the exact method for two related binomial proportions (Statxact; Cytel Statistical Software, Cambridge, MA).

### Sample size estimation

Because this study was a pilot study, sample size was not based on hypothesis testing, but instead on a reasonable estimation of the sample size necessary to allow us to look for trends when comparing data between the decision aid and control groups.

## Results

In accordance with our targeted enrollment and the capabilities of our single research assistant, we approached 212 of the 1247 potentially eligible patients who presented to our university internal medicine clinic for a return medical visit during our recruitment period (see Figure 1). One hundred twenty-nine agreed to participate; but 42 were determined to be ineligible based on their prior medical history or other eligibility criteria (17 had prior cardiovascular disease, 2 had other serious medical illness that made them a poor candidate for prevention, 11 reported they could not read because they were illiterate or did not have their glasses, 11 had no cholesterol checked in the last three years, and 1 had participated in the diabetes study in our clinic). There were no significant differences in the age and gender of those who participated and those who did not. Eighty-seven patients were enrolled in the study and randomized to the decision aid or usual care. Twelve patients (8 in the decision aid group and 4 in the control group) were subsequently determined to be ineligible with those in the decision aid group being slightly more likely to be male and younger. We, thus, included 75 patients in our final sample (41 in the decision aid group and 34 in the control group).

The baseline characteristics of included patients are shown in Tables 1 and 2. Participants in the decision aid and control groups were similar with regard to their demographic characteristics, self-perceived health status, comfort using a computer, accuracy in CHD risk perception, and their knowledge about effective prevention strategies. Participants in the decision aid group, however, had more CHD risk factors, a higher CHD risk, more current CHD interventions, more potential intervention options, a greater desire for independent decision making, and fewer concerns about determining the importance of lowering risk and communicating with their doctor than those in the control group.

### The effects of the decision aid on patient-provider discussions and patients' plans for CHD risk reduction

In unadjusted analysis (see Table 3), 16% (absolute difference; 95% CI -4 to +37%) more patients in the decision aid group talked with their provider about CHD risk reduction and 13% (absolute difference; 95% CI -7% to +34%) more had a specific plan to reduce their CHD risk. When plans were examined more broadly (e.g. including those who independently made a plan, but didn't talk with their doctor), results were similar (absolute difference +16%; 95% CI -1% to 33%). There were too few patients in our study to support adjusted analyses.

### Within group effects of the decision aid: patients' perceptions, their preferred participation in decision making, and their risk reduction choices

The decision aid did have a modest effect on patients' perception that CHD prevention requires a decision (see Table 4). Interestingly, there was no concomitant desire for increased participation in decision making about CHD with their doctor, but patients in the decision aid group did plan more interventions after using the decision aid (see Table 5).

## Discussion

Our study confirms prior reports that patients have limited knowledge about their CHD risk and the effective options for CHD risk reduction and provides new evidence that an individually-tailored computerized decision aid about CHD prevention appears to increase the proportion of patients who discuss CHD prevention with their doctors and the proportion of patients who have a specific plan for CHD risk reduction. Although results should be considered preliminary, the magnitude of the effect, if confirmed would be clinically important. Main findings were corroborated by within group increases in the perception that CHD prevention requires a personal decision and by within group increases in specific plans for CHD risk reduction.

Our finding of an increased proportion of patients with a specific plan for CHD risk reduction is consistent with findings from other decision aid studies promoting prevention and a choice among options to accomplish it. [19-22] This finding is also consistent with studies involving counseling about individualized CHD risk. For instance, in a study by Lovibond and colleagues [23], participants who received information on their individual CHD risk, behavioral options for intervention (e.g. dietary change, regular exercise, weight loss, and stress reduction), and personalized choice and goal setting, had greater reductions in CHD risk factors than those who received standard CHD health education. Given the similar content of our interventions, Lovibond and colleagues' finding offers hope that the plans patients expressed in our study might be reproducible (and significant in a larger study) and might additionally translate into actual CHD risk reduction.

There are a few important considerations in translation of patients' plans to actual CHD risk reduction, however. First, successful implementation of some of the options to lower CHD risk (e.g. hypertension medication, cholesterol medication) require doctor input. Users of our decision aid must therefore be able to successfully communicate their informed decision to their doctor and gain support for their chosen intervention. This was not true in the aforementioned study by Lovibond, in which all risk reduction strategies could be accomplished outside the clinical encounter. Whether our decision aid encourages users to successfully communicate their decisions to their doctor and gain the necessary support for intervention is a matter we didn't directly examine. We can not ignore the seeming paradox, however, that although decision aid users reported more discussions with their doctor and more plans for CHD risk reduction, they did not report a greater desire for shared decision making after using the decision aid. This issue bears further study to determine both its repeatability and causes, and whether we need to add a skill-building session [24,25] to our intervention to coach patients to interact with their doctor in order to lower their CHD risk. [26,27] Second, plans for CHD risk reduction do not ensure action and actual reduction in CHD risk. We have no guarantee that patients' chosen plans would actually help them circumvent their known barriers to CHD risk reduction. We are aware that patients are unlikely to adhere to risk reduction strategies without health system and social supports. [28] Therefore, we suspect that adjunctive adherence interventions may be needed to bring about actual CHD risk reduction. [29,30]

Even without modification, individually-tailored CHD decision aids clearly warrant further testing to address several research questions raised by our analysis. First, future studies should examine how overall CHD risk and the number of available CHD risk-reducing options affects patients' choices for risk reduction. Studies providing patients' global CHD risk estimates to doctors [31,32] show that doctors are more likely to prescribe risk reducing agents to patients at higher risk. Whether high risk patients choose more risk reduction options than low or moderate risk patients, however, remains to be seen. Second, future studies should assess the independent effect of CHD decision aids on patients and doctors. We didn't directly measure the effect of our decision aid on doctors' actions (i.e. via print-outs from the decision aid), but suspect this effect may be as important as increased patient requests for medication in producing actual risk reduction. Third, future studies should also determine the impact of the time spent with the decision aid. Differential time spent with the decision aid may imply differential processing of information or exploration of different information. Although our decision aid is largely constructed in linear fashion, patients accessed information on risk reduction strategies only by following individual links for each strategy; therefore, those who followed the links, rather than rushing forward with a priori knowledge to make decisions, may have made different choices. Finally, future studies should determine the impact of the decision aid in the absence of an active comparator. To the extent that physicians and patients in current clinical practice think about individual risk factors rather than combinations of multiple risk factors or global risk estimates, our use of a risk factor list as a comparator may underestimate the expected effects of the decision aid in actual clinical practice.

Future studies should also improve on the methodological challenges we encountered in our study. First, future studies should use a larger sample size: this would provide greater power to detect the effect of the decision aid, would improve the random distribution of baseline characteristics, and would also allow for adjustment of relevant confounders. In the current study, we were unable to adjust for important differences between the intervention and control groups (e.g. CHD risk, number of options for risk reduction, desire for independent decision making, and reported ease in communicating with their doctor), which may have biased the results toward a greater effect of the decision aid than was actually present. Second, future studies should consider random sampling and more dedicated study time to avoid attrition after randomization: these would help minimize selection bias. Although selection bias didn't appear to be a major problem in our study, we approached a convenience sample of patients and cannot exclude differences among our study sample and all of those eligible for inclusion. We also can't adequately estimate the effects of the small differences in the number of patients who left the intervention and control groups after randomization. Finally, future studies should consider both remote and practice-level randomization: this would reduce the possible contamination of results that could occur with local randomization techniques and doctors in the study who see both the decision aid and the control patients. The latter may have biased our results toward a lesser effect of the decision aid than was actually present.

## Conclusion

Our study takes an important first step toward defining the effect of an individually-tailored computerized decision aid on patients' plans to talk with their doctor about CHD prevention and intervene on their CHD risk. Future work should repeat these findings in a larger sample, addressing methodological and research issues raised in the current analysis. If decision aids can be shown to be repeatedly successful in increasing patients' plans for CHD risk reductions, the medical community will have an important new tool to aid the reduction of morbidity and mortality associated with CHD.

## Competing interests

Dr. Sheridan and Dr. Pignone have received consulting and licensing fees from Bayer, Inc. Dr. Simpson has received honoraria and consulting fees from Merck, Pfizer, and Galaxo Smith Kline and has received honoraria and grant funding from Schering Plough.

## Authors' contributions

SLS conceived of the study, participated in its design and coordination, directed statistical analysis, performed interpretation of data, and prepared the manuscript. MPP participated in the design of the study and helped with data interpretation. JS coordinated the study and collected the data. RJS participated in data interpretation. All authors reviewed the manuscript for important intellectual content and approved the final draft.

## Pre-publication history

The pre-publication history for this paper can be accessed here:



## Figures and Tables

**Figure 2:**
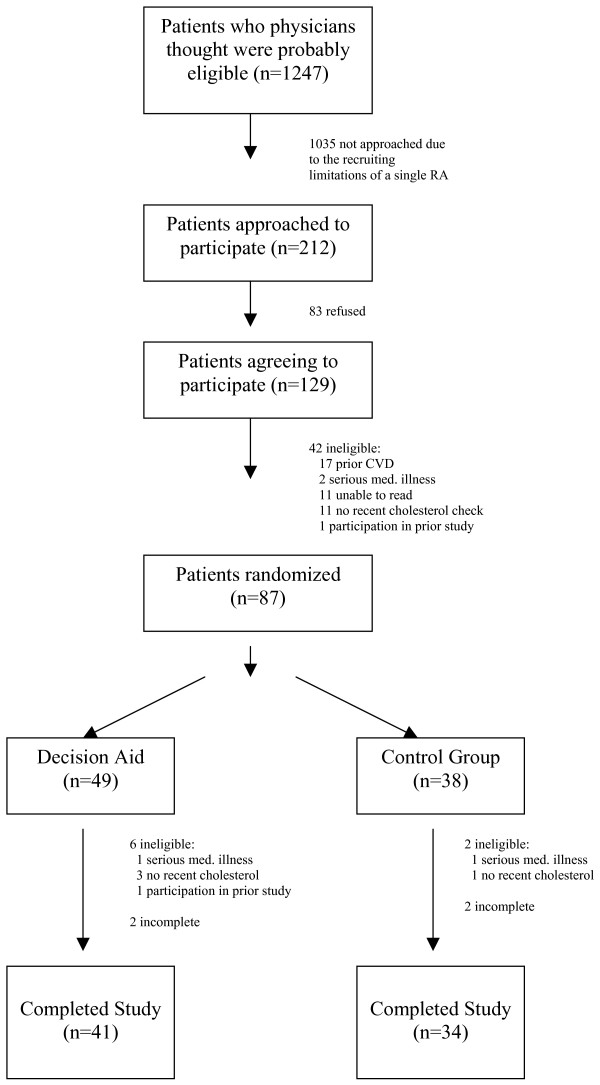
Recruitment and enrollment.

**Table 1:** Baseline participant characteristics

**Characteristic**	**Total Group**	**Control Group** **(n = 34)**	**Decision Aid Group** **(n = 41)**
Mean age (SD)	53 (9)	53 (11)	53 (8)
Female	59%	59%	59%
Race:			
White	73%	76%	71%
Black	23%	21%	24%
Education:			
At least some college	66%	71%	63%
Good self-perceived health status	67%	68%	66%
CHD Risk Factors			
BP > 140/90	39%	47%	31%
TC/HDL ratio > 4	43%	38%	46%
Smoker	39%	29%	46%
Diabetes	8%	6%	10%
Family History of CHD (age < 55)	31%	26%	34%
Actual CHD risk			
0–5%	43%	53%	34%
6–10%	25%	21%	27%
10–20%	24%	21%	29%
> 20%	5%	3%	7%
Current CHD Interventions:			
Blood pressure med	40%	38%	41%
Cholesterol med	19%	21%	17%
Smoking cessation	13%	6%	19%
Aspirin	39%	32%	44%
Diet low in saturated fat	48%	48%	49%
Exercise regularly	51%	41%	59%
# of Possible Intervention Options for CHD risk*:			
0	22%	24%	20%
1	23%	21%	25%
2	34%	42%	28%
3	15%	9%	20%
4	5%	3%	8%
Any Planned intervention, reasonable plans	68%	68%	68%
Any Planned intervention, best evidence interventions only*	56%	63%	51%
Preferred participation in decision making about CHD:			
I decide	12%	9%	15%
I decide, consider MD opinion	29%	26%	32%
Share decision	44%	50%	39%
MD decides, considers my opinon	7%	9%	5%
MD decides	7%	6%	7%
Comfort using computer	24%	24%	25%

*This includes hypertension med, cholesterol med, smoking cessation, aspirin.

**Table 2:** Baseline factors that might influence decision making

**Variable**	**Total Group** **(n = 75)**	**Control Group** **(n = 34)**	**Decision Aid Group** **(n = 41)**
**Perceived CHD Risk**			

Perceived CHD risk			
0–5%	33%	32%	34%
6–10%	32%	29%	34%
11–20%	12%	18%	12%
> 20%	20%	21%	20%

Accurate perception of global CHD risk	22%	23%	21%

**Knowledge about Effective Prevention Strategies**			

Identified "x" as strategy:			
HTN med	77%	80%	74%
Cholesterol med	59%	56%	61%
Smoking cessation	99%	100%	98%
Aspirin daily	68%	62%	73%
Diet low in saturated fat	83%	91%	76%
Exercise regularly	97%	97%	98%
Weight loss if overweight	92%	91%	93%
Reduce stress	88%	88%	88%
Drink a glass of red wine daily	39%	38%	39%
Take estrogen (women only)	12%	12%	12%
Take Vitamin E	17%	24%	12%
Take Calcium	11%	18%	5%
Monitor AM body temperature	4%	3%	5%

Accurately identified best strategies to lower CHD risk*	45%	47%	44%

**Perceived Barriers to CHD Risk Reduction**			

Lack of knowledge	82%	85%	81%
Difficulty determining how important it is to lower my risk	65%	76%	56%
Difficulty determining the best way to lower my risk	70%	79%	63%
Concerns about other health problems	57%	65%	51%
Lack of symptoms	58%	59%	56%
Difficulty communicating my needs to my doctor	43%	50%	37%

*This includes knowledge of all four interventions (e.g. hypertension med, cholesterol med, smoking cessation, aspirin) supported by the best evidence.

**Table 3:** Between group effects of the decision aid: Presence of patient-provider discussions and specific plans for CHD prevention

	**Control Group** **(n = 34)**	**Decision Aid Group** **(n = 41)**	**Absolute difference** (95% CI)*
CHD discussion with their doctor	24%	40%	16% (-4 to +37%)
Have a specific plan to reduce CHD risk and discuss it with their doctor	24%	37%	13% (-7% to +34%)
Have a specific plan to reduce CHD risk regardless of whether they discuss it with their doctor	74%	90%	16% (-1% to +33%)

*Pearson chi-square tests

**Table 4:** Within group effects of the decision aid

	**Baseline** **(n = 41)**	**Post-DA** **(n = 41)**	**Absolute Difference** (95% CI)*
**Effect on perception that CHD prevention requires a decision**			

Same way of lowering the chance of heart attack *is *right for everyone	40%	33%	-7% (-22% to 7%)
I am the only one who can decide how to best lower my chances of heart attack	30%	43%	+13% (-3% to 29%)

**Effect on desire to participate in decision-making**			

Preferred participation in decision making about CHD:			
I decide	15%	10%	-5% (-19% to 7%)
I decide, consider MD opinion	32%	28%	-5% (-19% to 7%)
Share decision	38%	38%	0% (-15% to 15%)
MD decides, considers my opinion	5%	10%	+5% (-7% to 19%)
MD decides	8%	13%	+5% (-7% to 19%)

* Exact binomial 95% confidence interval

**Table 5:** Within group effects: Planned risk reduction strategies across with the decision aid

**Planned CHD Interventions**	**In Decision Aid Group (n = 41)**		
	
	**Baseline**	**Post-DA**	Absolute Difference (95% CI) *
BP med, if HTN (n = 13)	8%	54%	+46% (9% to 75%)
Cholesterol med, If abnormal chol (n = 19)	5%	26%	+21% (-5% to 47%)
Smoking cessation, if smoking (n = 19)	21%	37%	+16% (-15% to 44%)
Aspirin, If high risk (> 10%; n = 17)	6%	53%	+47% (19% to 72%)
Diet low in saturated fat, all (n = 41)	27%	37%	+10% (-8% to 27%)
Exercise regularly, all (n = 41)	29%	34%	+5% (-12% to 21%)

*Exact binomial 95% CI
